# Comparing attenuation parameter and liver stiffness measurement using Hepatus and Fibroscan for the evaluation of steatosis and fibrosis in patients with chronic liver disease: A prospective study

**DOI:** 10.1371/journal.pone.0359831

**Published:** 2026-11-05

**Authors:** Cain-En Lim, Ram Prasad Sinnanaidu, Xin Tong Ng, Jin Ying Teh, Farahani Khamis, Anushya Vijayananthan, Wah-Kheong Chan

**Affiliations:** 1 Department of Biomedical Imaging, Faculty of Medicine, University of Malaya, Kuala Lumpur, Malaysia; 2 Gastroenterology and Hepatology Unit, Department of Medicine, Faculty of Medicine, University of Malaya, Kuala Lumpur, Malaysia; Kaohsiung Medical University Hospital, TAIWAN

## Abstract

**Background:**

There are limited studies comparing Hepatus (HP), a novel visual transient elastography device with Fibroscan (FS), one of the most studied and validated non-invasive tests in the field of hepatology.

**Methodology:**

HP and FS examinations were performed on patients with chronic liver disease by two operators, healthcare providers from different background, twice on each patient, at two different time points, independent of each other.

**Results:**

The data for 158 patients with 1264 examinations was analysed. There was moderate to strong correlation for attenuation parameter (Spearman’s rho 0.67, p < 0.001) and liver stiffness measurement (LSM) (Spearman’s rho 0.74, p < 0.001) obtained using the two devices. The mean difference (95% limits of agreement) in measurements was 15 (−69–99) dB/m for attenuation parameter and 1.2 (−5.0–7.2) kPa for LSM. There was fair to moderate agreement for diagnosis of steatosis grade >S0, > S1 and >S2 and substantial agreement for diagnosis of unlikely and likely compensated advanced chronic liver disease. The interquartile range (IQR) for attenuation parameter and IQR/median for LSM were lower for HP compared with FS (12 dB/m vs 33 dB/m, p < 0.001, and 13% vs 14%, p < 0.001, respectively). The intra- and inter-observer reliability using both devices were good to excellent with intraclass correlation coefficients of 0.86–0.97. Subgroup analyses demonstrated that agreement between HP and FS was maintained across obese and non-obese patients as well as patients with viral hepatitis and metabolic dysfunction-associated steatotic liver disease. HP had shorter examination time (43 s vs 62 s, p < 0.001) and fewer invalid measurements (0 vs 1, p < 0.001).

**Conclusion:**

HP demonstrated good comparability and reproducibility with FS for the assessment of hepatic steatosis and fibrosis, with shorter examination time and fewer invalid measurements.

## Introduction

Chronic liver disease represents a significant global health concern and remains a leading cause of death worldwide [1]. Early detection of liver fibrosis is critical in the management of chronic liver disease as it serves as a crucial predictor of long-term prognosis. Histopathological examination of liver biopsy specimen has been the gold standard for staging liver fibrosis. However, this invasive method has inherent limitations, including cost, a small risk of serious procedure-related side effects, sampling variability and observer variability. Due to these drawbacks, non-invasive diagnostic techniques such as transient elastography have been developed as alternatives.

Transient elastography conducted using Fibroscan (FS) generates shear waves from a vibrating device, which are measured by ultrasound [2]. This technique allows for simultaneous measurement of attenuation parameter and liver stiffness which correlates with steatosis and fibrosis, respectively. It has been extensively evaluated, leading to its integration into major clinical guidelines [3–5]. Visual transient elastography, conducted using Hepatus (HP), is a recent advancement in elastography technology. It integrates two-dimensional imaging and transient elastography into a single probe, allowing simultaneous visualization and quantitative assessment. In addition, it incorporates a dual-loop quality control mechanism to enhance stability and minimize the effect of subject’s respiration. However, there are limited studies comparing measurements obtained using the two devices. The aim of our study is to compare the attenuation parameter and liver stiffness measurement (LSM) obtained using HP and FS for assessing steatosis and fibrosis in patients with chronic liver disease. Furthermore, we aimed to evaluate intra- and inter-observer variability for measurements obtained using the two devices and to compare examination times.

## Methods

### Study design

This study adopted a cross-sectional framework similar to that described in our earlier publication on FibroTouch and Fibroscan [6]. This present work was conducted at the University of Malaya Medical Centre, Kuala Lumpur, Malaysia between September 2024 to March 2025. Inclusion and exclusion criteria followed the prior protocol: Adults (≥ 16 years) with chronic liver disease of any etiology were eligible, while individuals who were pregnant or had malignancy, ascites, or extrahepatic cholestasis were excluded. All procedures conformed to the ethical principles of the 1975 Declaration of Helsinki and were approved by the University Malaya Medical Centre Medical Research Ethics Committee (MECID:2024718-13926). Written informed consent was obtained from every participant.

A standardized data collection form was used to obtain demographic details, anthropometric indices, clinical characteristics, and laboratory results. BMI was determined by dividing body weight in kilogram by height squared (m^2^), with BMI ≥ 25 kg/m^2^ categorized as obese [7]. Waist circumference was measured at the midpoint between the lowest palpable rib and the iliac crest while standing upright. Central obesity was defined as a waist circumference ≥90 cm in men and ≥80 cm in women [8]. Venous blood was collected after an overnight fasting for liver function test, fasting plasma glucose, HbA1c, and lipid profile, performed within 6 months before or after elastography examination.

### Transient elastography

The overall scanning workflow were based on our previous work [6], however the present analysis introduced a new transient elastography device, the Hepatus 6 Diagnostic Ultrasound System (Shenzhen Mindray Bio-Medical Electronics Co. Ltd., China) for comparison with the Fibroscan 502 Touch (Echosens, France). Both devices were operated independently by two trained personnels: The first operator (RPS) was a doctor from the Gastroenterology and Hepatology Unit while the second operator (CEL) was from the Department of Biomedical Imaging. Each operator had completed more than 100 examinations before the commencement of the study. All measurements were performed after at least two hours of fasting. For every scan, a minimum of 10 valid readings was required. Repeated examinations were intentionally incorporated into the study design to evaluate intra- and inter-observer reproducibility. Each examination was performed independently by two trained operators who were blinded to previous measurement results, and examinations were not performed consecutively by the same operator to minimise potential measurement bias. Examination time and the number of invalid readings were recorded for subsequent comparison between the two devices.

### Fibroscan examinations

The examination protocol for FS followed the standard operating procedures reported in our previous study [6]. Both operators had substantial prior experience, having completed more than 100 FS scans before the start of the study. An examination was deemed successful when at least 10 valid measurements were obtained. Results were considered reliable if the IQR/median for LSM was ≤ 30% or when LSM was < 7.1 kPa even if IQR/median exceeded 30% [9,10]. In contrasts, a session was classified unsuccessful if fewer than 10 valid readings were achieved after 30 attempts [11]. The appropriate probe, M or XL, was automatically selected by the system’s built-in recommendation algorithm. The examination time, measured from the initial probe placement to the completion of the scan or termination of an unsuccessful attempt, was recorded for each assessment [11].

### Hepatus examinations

HP measurements were performed similarly to those used for FS, but examinations were performed using a single universal probe. Unlike with FS, neither operator had prior experience using the Hepatus device; both underwent specific training sessions and performed over 100 HP scans before study initiation. The criteria for determining successful, reliable, and unsuccessful examinations mirrored those used for FS. The total duration of each examination, measured from probe placement to completion or failure, was similarly documented.

### Statistical analysis

All analyses were conducted using a standard statistical software program, SPSS 29.0 (IBM SPSS Inc, Chicago, IL, USA). Continuous data were presented as mean ± standard deviation or median with interquartile range (IQR), where appropriate. Categorical variables were summarised as percentages. The relationship between LSM and attenuation parameters obtained from FS and HP was evaluated using Spearman’s rank correlation coefficient, rho (ρ) and visualised with scatter plots. Correlation strength was defined as follows: |ρ| ≤ 0.30 (weak), 0.30 < |ρ| < 0.70 (moderate), and |ρ| ≥ 0.70 (strong). Comparisons between devices for parameters such as median attenuation parameter value, IQR of attenuation parameter, LSM, IQR/median of LSM, examination duration and number of invalid readings were performed using the Wilcoxon signed-rank test. Statistical significance was defined as p < 0.05. Bland Altman analysis was applied to assess measurement agreement between the two elastography systems. Diagnostic concordance for steatosis and fibrosis, derived from attenuation parameter and LSM, was examined using Cohen’s Kappa (K) value. Interpretation of K followed standard categories: whereby K ≤ 0 (no agreement), 0.01–0.20 (slight), 0.21–0.40 (fair), 0.41–0.60 (moderate), 0.61–0.80 (substantial), and 0.81–0.99 (almost perfect agreement). Diagnostic thresholds for steatosis and fibrosis were based on previously validated Fibroscan studies. The attenuation parameter cut-offs for steatosis grades >S0, > S1 and >S2 were 248 dB/m, 268 dB/m and 280 dB/m, respectively [12]. For LSM, values of <10 kPa and >15 kPa were used to rule out or confirm compensated advanced chronic liver disease (cACLD) [3,13]. The diagnostic performance of HP relative to FS was assessed by calculating the area under the receiving operating characteristic curve (AUROC), categorised as: 0.90–1.00 = excellent, 0.80–0.90 = good, 0.70–0.80 = fair, < 0.70 = poor. Because repeated examinations were performed to assess measurement reproducibility, intra- and inter-observer reliability were analysed using the intraclass correlation coefficient (ICC), interpreted as: 0.90–1.00 = excellent, 0.80–0.90 = good, 0.70–0.80 = fair, and <0.70 = poor. In addition, subgroup analyses were performed according to obesity status and disease etiology [viral hepatitis versus metabolic dysfunction-associated steatotic liver disease (MASLD)]. Exploratory patient-level correlation analyses were also performed between LSM obtained using HP and FS and available serum biochemical markers (i.e., aspartate aminotransferase [AST], alanine aminotransferase [ALT] and gamma-glutamyl transferase [GGT]). To account for repeated examinations within the same participant, the four LSM measurements obtained with each device were averaged for each participant. Correlations were assessed using Spearman’s rank correlation coefficient.

## Results

A total of 158 patients with chronic liver disease were enrolled in this prospective study. Patient characteristics are summarised in Table 1. The median age of the cohort was 62 (52–67) years, with 47.5% being male. The median BMI was 25.9 (23.6–28.6) kg per m^2^ and and median waist circumference was 90.5 (82.4–98.7) cm. Obesity and central obesity were identified in 57% and 63.9% of participants, respectively. Comorbidities were prevalent in this cohort, with 57 patients (36.1%) having diabetes mellitus, 80 patients (50.6%) having hypertension, and 96 patients (60.8%) having dyslipidaemia. The most predominant etiology of chronic liver disease was MASLD (57%), followed by chronic hepatitis B (31.6%), chronic hepatitis C (2.5%) and others (15.2%). Both HP and FS achieved a 100% success and reliability rate. Among FS assessments, 22 (3.5%) required the XL probe whereas all HP examinations were completed using a single universal probe.

**Table 1:** Patient characteristics.

	Overall population (n = 158)
Age, years	62 (52–67)
Male, n (%)	75 (47.5)
Body mass index, kg/m²	25.9 (23.6–28.6)
Waist circumference, cm	90.5 (82.4–98.7)
Obesity, n (%)	90 (57)
Central obesity, n (%)	101 (63.9)
Diabetes mellitus, n (%)	57 (36.1)
Hypertension, n (%)	80 (50.6)
Dyslipidaemia, n (%)	96 (60.8)
Systolic blood pressure, mmHg	138 (125–146)
Diastolic blood pressure, mmHg	75 (68–80)
Albumin, g/L	39 (38–42)
Bilirubin, µmol/L	15 (10–17)
ALT, U/L	40 (18–51)
ALP, U/L	94 (65–102)
GGT, U/L	37 (20–78)
Total Cholesterol, mmol/L	4.9 (3.5–5.6)
HDL Cholesterol, mmol/L	1.3 (1.1–1.5)
LDL Cholesterol, mmol/L	2.9 (1.7–3.6)
Triglyceride, mmol/L	1.6 (1.0–1.8)
Fasting blood glucose, mmol/L	6.1 (4.9–6.8)
HbA1c, %	6.4 (5.6–7.0)
Aetiology of chronic liver disease:	
Chronic hepatitis B, n (%)	50 (31.6)
Chronic hepatitis C, n (%)	4 (2.5)
MASLD, n (%)	90 (57)
Others, n (%)	24 (15.2)

Obesity was defined as body mass index ≥25 kg per m^2^.

Central obesity was defined as waist circumference > 90 cm for men and > 80 cm for women.

**ALT**, alanine aminotransferase; **ALP**, alkaline phosphatase; **GGT**, Y-glutamyl transpeptidase; **LDL**, low density lipoprotein; **HDL**, high density lipoprotein; **MASLD**, metabolic dysfunction-associated steatotic liver disease.

### Attenuation parameter

Paired measurements were obtained from 632 examinations performed on 158 patients using both HP and FS, with no adverse events recorded for either procedure. There was a moderate correlation between HP and FS for attenuation parameter measurements (Spearman’s rho = 0.67, p < 0.001) (Fig 1a). The relationship between attenuation parameter measured by the two devices relative to their mean values is illustrated in Fig 1b. Overall, attenuation parameter obtained using HP were significantly lower compared with those obtained using FS (Table 2). In addition, the IQR for attenuation parameter obtained using HP was also significantly lower than that obtained using FS (Table 2). Bland–Altman analysis revealed a mean difference of 15 dB/m between attenuation parameters obtained using HP and FS, with the 95% limit of agreement ranging from −69 dB/m and 99 dB/m (Fig 1c). When attenuation parameters were categorised according to steatosis grades, moderate agreement between the two devices was observed for the diagnosis of steatosis grade >S0 and >S1, with Cohen’s kappa value of 0.55 and 0.49, respectively. Fair agreement was observed for the diagnosis of steatosis grade >S2 with Cohen’s kappa value of 0.40. When using FS as the reference standard, attenuation parameters measured by HP demonstrated good diagnostic performance for detecting steatosis grade > S0, with an AUROC of 0.85.

**Table 2:** Attenuation parameter, interquartile range for attenuation parameter, liver stiffness measurement and interquartile range /median for liver stiffness measurement obtained using Hepatus and Fibroscan.

	Hepatus, n = 632	Fibroscan, n = 632	p
Attenuation parameter, dB/m	255 (224–287)	269 (228–313)	<0.001
IQR, dB/m	12 (7–16)	33 (20–42)	<0.001
LSM, kPa	7.2 (4.5–8.1)	8.4 (4.8–9.4)	<0.001
IQR/median, %	13 (8–17)	14 (9–18)	0.001

The median attenuation parameter, IQR of attenuation parameter, LSM, and IQR/median of LSM obtained using the two devices were compared using Wilcoxon test. Significance was assumed when p < 0.05.

**IQR**, interquartile range; **LSM**, liver stiffness measurement.

**Fig 1:**
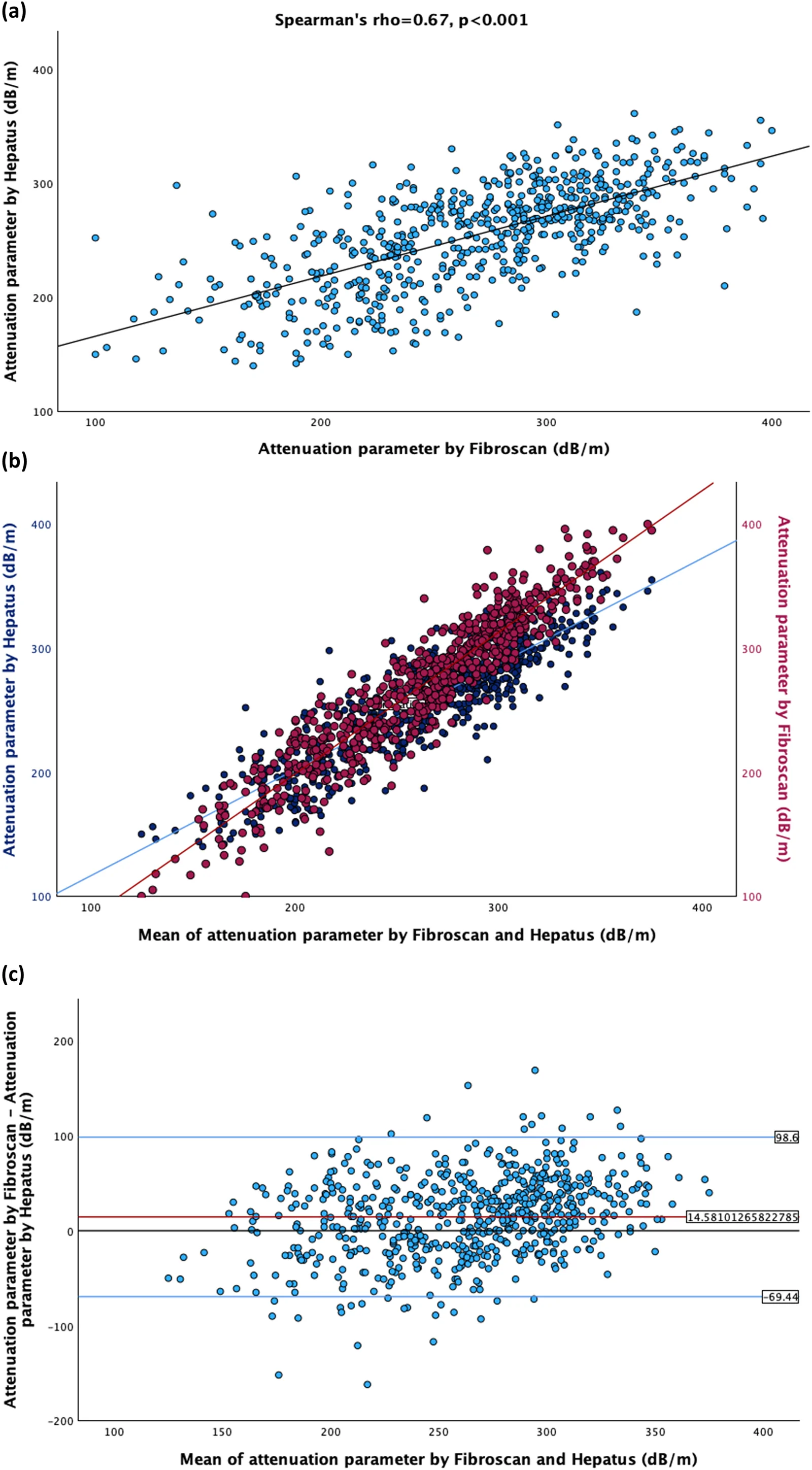
Comparison of attenuation parameter obtained using Hepatus and Fibroscan. (a) Scatter plot showing attenuation parameter obtained using Hepatus plotted against attenuation parameter obtained using Fibroscan (b) Scatter plot showing attenuation parameter obtained using Hepatus and Fibroscan plotted against mean attenuation parameter obtained using the two devices (c) Bland–Altman plot showing mean difference and 95% limit of agreement for attenuation parameter obtained using Hepatus and Fibroscan.

Operator variability analysis demonstrated strong correlations between Operator 1 and Operator 2 for attenuation parameter measurements with both devices (HP: Spearman’s rho = 0.70, p < 0.001, Fig 2a; FS: Spearman’s rho = 0.80, p < 0.001, Fig 2b). The intra-observer reliability of attenuation parameter measurements was good to excellent for both devices. For HP, the ICC was 0.93 (95% CI, 0.91–0.95) for Operator 1 and 0.86 (95% CI, 0.81–0.90) for Operator 2. Similarly, for FS, the ICC was 0.89 (95% CI, 0.85–0.92) for Operator 1 and 0.91 (95% CI, 0.88–0.93) for Operator 2. The inter-observer reliability of attenuation parameter measurements was also good for both devices, with ICC values of 0.82 (95% CI, 0.77–0.87) for HP and 0.89 (95% CI, 0.86–0.91) for FS.

**Fig 2:**
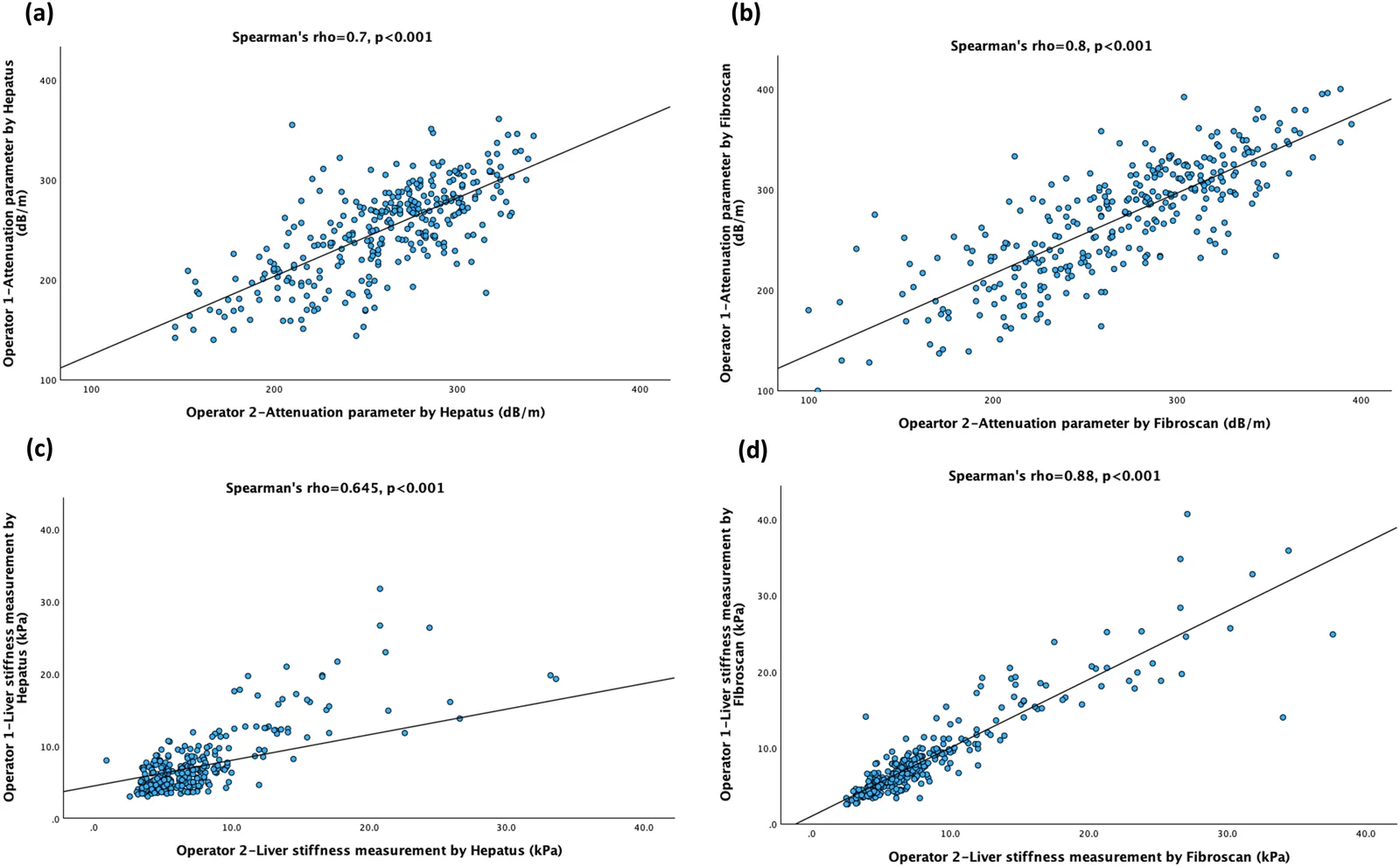
Comparison of attenuation parameter and liver stiffness measurement obtained by two operators when using Hepatus and when using Fibroscan. (a) Scatter plot showing attenuation parameter obtained using Hepatus by Operator 1 plotted against attenuation parameter obtained using Hepatus by Operator 2. (b) Scatter plot showing attenuation parameter obtained using Fibroscan by Operator 1 plotted against attenuation parameter obtained using Fibroscan by Operator 2. (c) Scatter plot showing liver stiffness measurement obtained using Hepatus by Operator 1 plotted against liver stiffness measurement obtained using Hepatus by Operator 2. (d) Scatter plot showing liver stiffness measurement obtained using Fibroscan by Operator 1 plotted against liver stiffness measurement obtained using Fibroscan by Operator 2.

### Liver stiffness measurement

The median LSM obtained using HP was 7.2 (4.5–8.1) kPa, which was significantly lower than that obtained using FS at 8.4 (4.8–9.4) kPa (p < 0.001) (Table 2). The IQR/median ratio for LSM was also significantly lower with HP [13 (8–17) %] compared with FS [14 (9–18) %] (p = 0.001) (Table 2). There was a strong correlation between HP and FS for LSM (Spearman’s rho = 0.74, p < 0.001) (Fig 3a). The relationship between LSM measurements obtained using the two devices relative to their mean values is illustrated in Fig 3b. Agreement between the two devices was further evaluated using Bland–Altman analysis, which revealed a mean difference of 1.2 kPa between HP and FS, with the 95% limits of agreement ranging from −5.0 to 7.2 kPa (Fig 3c). There was substantial agreement between the two devices for excluding and diagnosing compensated advanced chronic liver disease, with Cohen’s kappa value of 0.69 and 0.64, respectively. When using FS as reference standard, LSM measured with HP demonstrated excellent diagnostic performance, with AUROC values of 0.91 for excluding cACLD and 0.96 for diagnosing cACLD.

**Fig 3:**
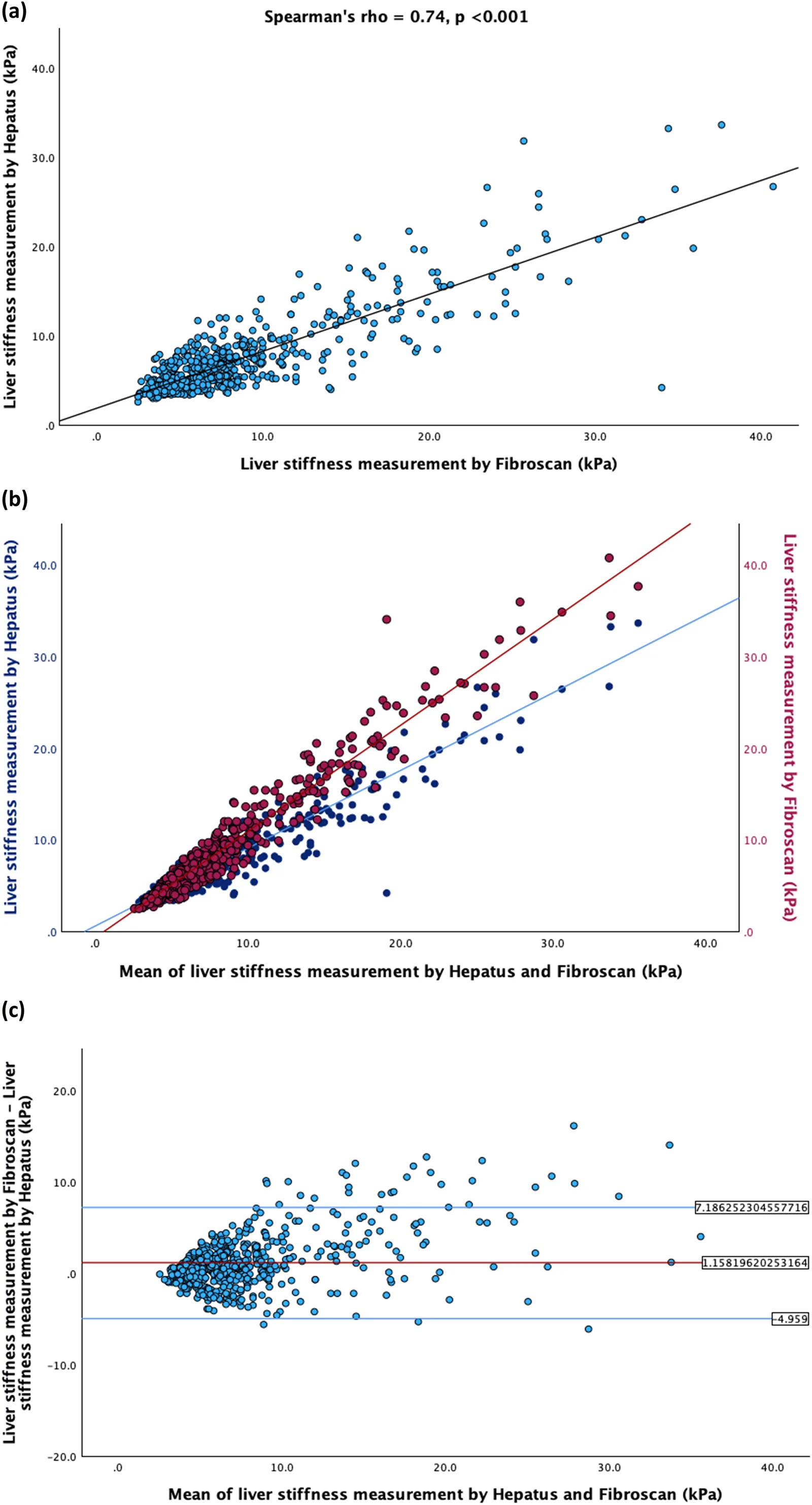
Comparison of liver stiffness measurement obtained using Hepatus and Fibroscan. (a) Scatter plot showing liver stiffness measurement obtained using Hepatus plotted against liver stiffness measurement obtained using Fibroscan (b) Scatter plot showing liver stiffness measurement obtained using Hepatus and Fibroscan plotted against mean liver stiffness measurement obtained using the two devices (c) Bland–Altman plot showing mean difference and 95% limit of agreement for liver stiffness measurement obtained using Hepatus and Fibroscan.

Inter-operator reproducibility was evaluated by comparing measurements from the two independent operators. There was moderate to strong correlation between Operator 1 and Operator 2 for LSM measured using HP (Spearman’s rho = 0.65, p < 0.001, Fig 2c) and FS (Spearman’s rho = 0.88, p < 0.001, Fig 2d). The intra-observer reliability of LSM was excellent for both devices. For HP, the ICC was 0.95 (95% CI, 0.93–0.96) for Operator 1 and 0.97 (95% CI, 0.96–0.98) for Operator 2. Similarly, FS demonstrated excellent intra-observer reliability with ICC values of 0.95 (95% CI, 0.93–0.96) for Operator 1 and 0.94 (95% CI, 0.92–0.96) for Operator 2. The inter-observer reliability for LSM was good to excellent, with ICC values of 0.89 (95% CI, 0.86–0.91) for HP and 0.95 (95% CI, 0.94–0.96) for FS.

### Time taken for an examination and number of invalid measurements

In the overall population, HP demonstrated a significantly shorter examination time compared to FS across all subgroups (Table 3). The median examination time was 43 (20–53) seconds for HP versus 62 (31–64) seconds for FS (p < 0.001). The number of invalid measurements was 0 for HP whereas FS recorded a median of 1 (0–2) invalid measurements (p < 0.001). Despite the higher number of invalid individual measurements observed with Fibroscan, all examinations in both groups ultimately achieved the predefined criterion of at least 10 valid measurements. Consequently, the overall examination success rate was 100% for both Hepatus and Fibroscan. When stratified according to obesity status, HP consistently demonstrated shorter examination times and fewer invalid measurements. In non-obese patients, the median examination time was 42 (21–53) seconds for HP and 56 (29–58) seconds for FS (p < 0.001), with invalid measurements of 0 and 1 (0–1), respectively (p < 0.001). In obese patients, the examination time remained shorter with HP (44 [20–53] seconds) compared with FS (65 [33–70] seconds, p < 0.001), while invalid measurements were 0 with HP and 2 (0–2) with FS (p < 0.001).

### Subgroup analyses according to obesity status and disease etiology

Among obese patients, there was moderate correlation between HP and FS for attenuation parameter (Spearman’s rho = 0.59, p < 0.001) and strong correlation for LSM (Spearman’s rho = 0.82, p < 0.001). Among non-obese patients, strong correlations were observed for attenuation parameter (Spearman’s rho = 0.78, p < 0.001) and LSM (Spearman’s rho = 0.76, p < 0.001) (S1 Table). When stratified according to disease etiology, strong correlations were observed between HP and FS among patients with viral hepatitis (attenuation parameter Spearman’s rho = 0.76, LSM Spearman’s rho = 0.81; both p < 0.001), whereas for patients with MASLD, moderate correlation was observed for attenuation parameter (Spearman’s rho = 0.65, p < 0.001) and strong correlation was observed for LSM (Spearman’s rho = 0.79, p < 0.001) (S2 Table).

### Correlation with serum biochemical markers

Exploratory patient-level analyses demonstrated significant positive correlations between AST and LSM measured using HP (Spearman’s rho = 0.297, p < 0.001) and FS (rho = 0.381, p < 0.001). ALT was also significantly correlated with LSM measured using HP (rho = 0.201, p = 0.012) and FS (rho = 0.251, p = 0.002), as was GGT (HP: rho = 0.320, p < 0.001; FS: rho = 0.416, p < 0.001) (S3 Table).

**Table 3:** Time taken for an examination and number of invalid measurements for Hepatus and Fibroscan in the overall population and when stratified according to obese and non-obese groups.

	Hepatus	Fibroscan	p
**Overall**	**n = 632**	**n = 632**	
Time taken for an examination, s	43 (20–53)	62 (31–64)	<0.001
Number of invalid measurements	0	1 (0–2)	<0.001
**Non-obese**	**n = 272**	**n = 272**	
Time taken for an examination, s	42 (21–53)	56 (29–58)	<0.001
Number of invalid measurements	0	1 (0–1)	<0.001
**Obese**	**n = 360**	**n = 360**	
Time taken for an examination, s	44 (20–53)	65 (33–70)	<0.001
Number of invalid measurements	0	2 (0–2)	<0.001

The time taken for an examination and number of invalid measurements using the two devices were compared using Wilcoxon test. Significance was assumed when p < 0.05.

Obesity was defined as BMI ≥ 25 kg per m^2^.

## Discussion

There are limited studies comparing HP and FS. Most studies to date originate from China, where HP technology was initially developed [14–16]. Of those reports, one focused on attenuation parameter in patients with chronic hepatitis B, while the other two evaluated LSM in patients with chronic liver disease. To date, only one study was conducted outside China, comparing HP and FS for evaluating fibrosis among patients with chronic hepatitis B [17]. Our study represents an additional study from outside China and extends previous findings by comparing both attenuation parameter and LSM obtained using HP with those obtained using FS among patients with chronic liver disease. Moreover, our study is the first known study to evaluate intra- and inter-observer reliability of attenuation parameter and LSM obtained by HP when performed by operators from different professional backgrounds.

Our data confirmed strong inter-device correlation for LSM. Although HP consistently yielded lower LSM values than FS, there was substantial agreement for excluding and diagnosing compensated advanced chronic liver disease using 10 kPa and 15 kPa cut-offs, respectively. The mean device difference of 1.2 kPa between LSM obtained using HP and FS is generally considered to be clinically acceptable. Operator-related and patient-related factors can produce significant variations in LSM obtained using transient elastography that are unrelated to disease severity [18]. LSM performed at different intercostal sites have also been shown to produce significant within-individual variability, particularly in individuals with class 3 obesity [19]. Only differences of 20–30% or more in LSM are considered clinically significant [19,20].

Our study showed moderate correlation between attenuation parameter obtained using HP and FS. Although attenuation parameter values were lower when obtained using HP compared with FS, there was moderate agreement between HP and FS for the diagnosis of steatosis grades >S0 and >S1 and fair agreement for the diagnosis of steatosis grade >S2. The mean difference of 15 dB/m between attenuation parameter obtained using HP and FS is not likely to be clinically significant. The accuracy of attenuation parameter for the diagnosis of hepatic steatosis has been shown to be greater when the IQR of CAP is < 30 dB/m [21,22]. In our study, the overall IQR of attenuation parameter obtained using HP is 12 dB/m. Overall, we found that HP produced more consistent result with significantly lower IQR for attenuation parameter and IQR/median for LSM compared with FS.

The present study was designed to evaluate agreement between HP and FS rather than to establish the diagnostic accuracy of HP against independent reference standards. Although FS is an extensively validated and widely adopted non-invasive modality for assessing hepatic fibrosis and steatosis, agreement with FS should not be interpreted as direct validation against liver histology or advanced imaging techniques. Liver biopsy is considered the reference standard for the assessment of hepatic steatosis and fibrosis but was not performed because of its invasive nature and inherent limitations, including sampling and observer variability. Magnetic resonance imaging-proton density fat fraction (MRI-PDFF) and magentic resonance elastography (MRE) were also not routinely available at our institution.

Exploratory analyses demonstrated modest positive correlations between AST, ALT and GGT and LSM obtained using both HP and FS. These findings provide additional comparative information but should not be interpreted as diagnostic validation, as routine serum biochemical markers are not independent reference standards for liver fibrosis. Correlations between these biochemical markers and attenuation parameter were not performed because routine liver biochemical markers are not validated measures of hepatic steatosis.

Additional subgroup analyses demonstrated that agreement between HP and FS was maintained across clinically relevant patient populations, including obese and non-obese individuals as well as patients with viral hepatitis and MASLD. Although correlations for attenuation parameter were somewhat lower among obese patients and those with MASLD, correlations for LSM remained consistently strong across all subgroups. These findings support the robustness of the agreement between HP and FS across different patient populations commonly encountered in clinical practice.

One of the key findings of this study was the high intra- and inter-observer reliability of both HP and FS when performed by operators from different professional backgrounds, suggesting that adequate training, regardless of clinical specialty, is sufficient to achieve reproducible results. Although inter-operator correlation for LSM obtained using HP was lower than that observed with FS, inter-observer reliability assessed using ICC remained good to excellent. The lower correlation observed may reflect the relative novelty of the HP system and an operator learning curve. Nevertheless, the consistently high ICC values indicate that reliable measurements can be achieved with adequate training. We have previously reported a similar observation when comparing FS performance across personnel of different expertise [11]. In the current study, we also observed that HP produced fewer invalid measurements and required shorter examination time than FS. The use of single universal probe in HP, in contrast to the two-probe system in FS, is likely to have contributed to both of these advantages. The XL probe in FS was specifically introduced to mitigate failed and unreliable examinations using the conventional M probe, especially in obese patients [23]. HP’s avoidance of this limitation through a universal probe is a practical benefit, particularly in populations with high prevalence of obesity.

Despite our best effort, this study has several limitations. First, we did not perform liver biopsy on patients included in the study to confirm their grade of steatosis and stage of fibrosis. Although liver biopsy has been the reference standard for evaluating diagnostic tests, it is invasive and associated with a small risk of serious complications, including death. Furthermore, it has inherent limitations of sampling and observer variability. Importantly, liver biopsy is less and less performed nowadays with the increasing use of simple and reliable non-invasive tests, including FS, which is one of the most studied and validated non-invasive tests in the field of hepatology. Secondly, as this is a single centre study in a hospital setting, the findings may not be generalised to other populations. Thirdly, we did not use the latest FS device (e.g., Fibroscan 630 Expert) because the device was not yet available at the time of the study. In addition, multiple elastography examinations were performed for each participant. This design was intentionally adopted to evaluate intra- and inter-observer reproducibility. However, repeated measurements obtained from the same participant within a relatively short time frame may introduce bias and this should be considered when interpreting the findings. Nevertheless, we have ensured that repeated examinations were not performed consecutively by the same operator and each examination was performed independently by two operators who were blinded to the result of previous measurement. Exploratory correlations with available serum biochemical markers were performed; however, validated composite fibrosis indices such as Fibrosis-4 index could not be evaluated because platelet count was not collected in the original study dataset. Furthermore, the absence of independent reference standards such as liver histology, MRE or MRI-PDFF limits the assessment of the diagnostic accuracy of HP beyond its comparison with FS.

In conclusion, HP demonstrated moderate-to-strong correlation with FS for attenuation parameter and LSM, with good-to-excellent intra- and inter-observer reliability. HP also had shorter examination times and fewer invalid measurements. These findings demonstrate good comparability and reproducibility between HP and FS for the assessment of hepatic steatosis and fibrosis in patients with chronic liver disease.

## Supporting information

S1 Table Correlation between Hepatus and Fibroscan according to obesity status. (DOCX)

S2 Table Correlation between Hepatus and Fibroscan according to disease etiology. (DOCX)

S3 Table Correlation between liver stiffness measurements obtained using Hepatus and Fibroscan and serum biochemical markers. (DOCX)

S1 File Study dataset. (XLSX)
