# Supplementary material for: Comparing attenuation parameter and liver stiffness measurement using Hepatus and Fibroscan for the evaluation of steatosis and fibrosis in patients with chronic liver disease: A prospective study
Source: PLoS One. 2026 Nov 5;21(11):e0359831. doi: 10.1371/journal.pone.0359831 (PMC13739124; doi:10.1371/journal.pone.0359831)
Supplement: Supplementary file 1 [file pone.0359831.s001.docx]

**Supplementary Table S1** Correlation between Hepatus and Fibroscan according to obesity status

|  | n | Spearman’s rho for attenuation parameter | p-value | Spearman’s rho for liver stiffness measurement | p-value |
| --- | --- | --- | --- | --- | --- |
| Obese | 90 | 0.587 | <0.001 | 0.819 | <0.001 |
| Non-obese | 68 | 0.779 | <0.001 | 0.758 | <0.001 |
