# Supplementary material for: Comparing attenuation parameter and liver stiffness measurement using Hepatus and Fibroscan for the evaluation of steatosis and fibrosis in patients with chronic liver disease: A prospective study
Source: PLoS One. 2026 Nov 5;21(11):e0359831. doi: 10.1371/journal.pone.0359831 (PMC13739124; doi:10.1371/journal.pone.0359831)
Supplement: Supplementary file 2 [file pone.0359831.s002.docx]

**Supplementary Table S2** Correlation between Hepatus and Fibroscan according to disease etiology

|  | n | Spearman’s rho for attenuation parameter | p-value | Spearman’s rho for liver stiffness measurement | p-value |
| --- | --- | --- | --- | --- | --- |
| Viral hepatitis | 54 | 0.758 | <0.001 | 0.808 | <0.001 |
| MASLD | 90 | 0.652 | <0.001 | 0.788 | <0.001 |

*MASLD, metabolic dysfunction-associated steatotic liver disease*
