# Supplementary material for: Comparing attenuation parameter and liver stiffness measurement using Hepatus and Fibroscan for the evaluation of steatosis and fibrosis in patients with chronic liver disease: A prospective study
Source: PLoS One. 2026 Nov 5;21(11):e0359831. doi: 10.1371/journal.pone.0359831 (PMC13739124; doi:10.1371/journal.pone.0359831)
Supplement: Supplementary file 3 [file pone.0359831.s003.docx]

**Supplementary Table S3** Correlation between liver stiffness measurements obtained using Hepatus and Fibroscan and serum biochemical markers

| Serum biochemical marker | Hepatus LSM, Spearman’s rho | p-value | FibroScan LSM, Spearman’s rho | p-value |
| --- | --- | --- | --- | --- |
| Aspartate aminotransferase | 0.297 | <0.001 | 0.381 | <0.001 |
| Alanine aminotransferase | 0.201 | 0.012 | 0.251 | 0.002 |
| Gamma-glutamyl transferase | 0.320 | <0.001 | 0.416 | <0.001 |

*LSM, liver stiffness measurement*
