# Supplementary material for: Comparing attenuation parameter and liver stiffness measurement using Hepatus and Fibroscan for the evaluation of steatosis and fibrosis in patients with chronic liver disease: A prospective study
Source: PLoS One. 2026 Nov 5;21(11):e0359831. doi: 10.1371/journal.pone.0359831 (PMC13739124; doi:10.1371/journal.pone.0359831)
Supplement: Supplementary file 4 [file pone.0359831.s006.docx]

Reviewers' Comments to Author:
Reviewer: 1

This manuscript presents a comparison between Hepatus, a relatively new transient elastography device, and FibroScan for the assessment of hepatic steatosis and fibrosis in patients with chronic liver disease. The study addresses a clinically relevant topic, as the development of reliable and accessible non-invasive tools for liver disease assessment remains an important unmet need. The authors should be commended for the substantial effort involved in conducting repeated examinations by different operators and evaluating both intra- and inter-observer reliability. However, several major concerns regarding the study design, statistical analysis, and validation strategy limit the interpretation of the findings. Most importantly, the handling of repeated measurements raises concerns regarding the independence of observations, and the comparison with FibroScan alone is insufficient to establish the clinical validity of Hepatus as a tool for assessing liver fibrosis and steatosis. Furthermore, additional analyses are required to better characterize the performance of Hepatus across clinically relevant subgroups and in relation to established non-invasive biomarkers. Therefore, I recommend Major Revision. My specific comments are provided below.

1) This manuscript analyzes 1,264 examinations performed in 158 individuals. This corresponds to eight examinations per subject, assuming that two examiners each performed two different tests twice. While the substantial effort involved in collecting these data is commendable, there are statistical concerns regarding the treatment of all examination results as independent observations. Measurements obtained repeatedly from the same individual should not be considered independent variables; rather, they should be averaged or otherwise appropriately accounted for as repeated measures. In addition, the reproducibility of the measurements obtained from repeated examinations in the same patient should be evaluated and reported.

We thank the reviewer for this important comment. The repeated examinations were intentionally incorporated into the study design to evaluate both intra- and inter-observer reproducibility of the two devices. Each examination was performed independently by two trained operators who were blinded to the results of previous measurements, and examinations were not performed consecutively by the same operator.

We agree that repeated measurements obtained from the same participant are not fully independent observations. However, our primary objective was to compare device performance under routine clinical conditions and to assess measurement reproducibility. To address this concern, we have clarified the study design and statistical approach in the revised manuscript and emphasised that reproducibility was formally evaluated using intra-observer and inter-observer intraclass correlation coefficients (ICC), which demonstrated good to excellent reliability for both Hepatus and FibroScan.

We have also acknowledged in the limitations section that repeated measurements from the same individual may introduce bias and should be considered when interpreting the findings.

(page 5 line 100-104; page 7 line 148-149; page 17 line 352-361)

2) FibroScan has been extensively validated against established reference standards, including liver biopsy, for the assessment of hepatic fibrosis and steatosis. In contrast, as the authors themselves acknowledge, Hepatus is a relatively new technique for which robust clinical evidence has not yet been established. Furthermore, FibroScan provides only an indirect assessment of liver fibrosis and steatosis. Therefore, even if Hepatus demonstrates a correlation with FibroScan measurements, this does not necessarily indicate a direct correlation with the actual degree of fibrosis or steatosis. Before comparisons with FibroScan can be considered sufficient validation, the relationship between Hepatus and established reference standards, such as liver biopsy, MRI elastography, and MR spectroscopy, should be investigated.

We thank the reviewer for this insightful comment and agree that correlation with FibroScan does not establish direct correlation with histological fibrosis or steatosis. The objective of the present study was not to validate Hepatus against histological or advanced imaging reference standards, but rather to compare its measurements with those obtained using FibroScan, which is one of the most extensively studied and widely adopted non-invasive modality for liver fibrosis and steatosis assessment that has been incorporated into major international guidelines.

We acknowledge that liver biopsy remains the reference standard for fibrosis and steatosis assessment. However, liver biopsy has its inherent limitations, including its invasive nature, small risk of serious complications, limited acceptability, sampling variability and observer variability. Moreover, the field is moving away from liver biopsy and is increasingly relying on non-invasive tests. Therefore, biopsy was not performed solely for the purpose of this study due to ethical reasons.

On the other hand, MRI elastography and MR-based fat quantification are not routinely available in our institution. We have revised the Discussion section to further emphasise that our findings demonstrate agreement between Hepatus and FibroScan rather than direct validation against histological or advanced imaging reference standards. Future studies comparing Hepatus with liver biopsy, MRI elastography, and MRI-PDFF would be valuable.

(page 15 line 309-320)

3) The correlation between Hepatus and FibroScan measurements should be evaluated separately in relevant subgroups, including obese versus non-obese individuals and patients with viral versus non-viral liver disease.

We thank the reviewer for this valuable suggestion. Additional subgroup analyses were performed according to obesity status and disease etiology using patient-level averaged measurements.

Among obese patients (n = 90), the correlation between Hepatus and FibroScan was moderate for attenuation parameter (Spearman’s rho = 0.59) and strong for liver stiffness measurement (rho = 0.82). Among non-obese patients (n = 68), the correlation remained strong for attenuation parameter (rho = 0.78) and liver stiffness measurement (rho = 0.76).

When stratified according to disease etiology, strong correlations were observed in patients with viral hepatitis (attenuation parameter rho = 0.76; liver stiffness rho = 0.81), whereas for patients with MASLD, moderate correlation was observed for attenuation parameter (rho = 0.65) and strong correlation was observed for liver stiffness (rho = 0.79).

These findings support the robustness of the agreement between Hepatus and FibroScan across clinically relevant patient subgroups. The results have been added as a supplementary table in the revised manuscript.

(page 7 line 149-151; page 8 line 163-172; page 16 line 321-327)

4) In addition, correlation analyses should be performed between both FibroScan and Hepatus measurements and established non-invasive serum biomarkers (AST, ALT and so on) and indices, such as the FIB-4 index. Such analyses would provide further insight into the clinical validity and utility of these modalities for the assessment of liver fibrosis.

We thank the reviewer for this suggestion. We agree that correlation with established non-invasive fibrosis biomarkers such as FIB-4 would provide additional information regarding clinical validity and utility. However, these analyses were not part of the original study objectives and were therefore not included. We have acknowledged this as a limitation of the present study and have highlighted it in the revised manuscript. Future studies evaluating Hepatus alongside established serum-based fibrosis markers would be valuable.

(page 17 line 358-361)

Reviewer 2

This study compares attenuation parameter (AP) and liver stiffness (LS) measurements with Fibroscan (FS) and Hepatus (HP). The authors enrolled 158 chronic liver disease patients. Two operators in different backgrounds performed twice for each patient at two different points. The authors analyzed 1264 exams and showed moderate correlations in AP and LS between FS and HP. The mean difference of AP and LS were15 (-69-99) dB/m and 1.2 (-5.0-7.2) kPa. There were also agreement in the steatosis grade and diagnosis of compensated advanced chronic liver disease (cACLD). While both HP and FS were in good intra- and inter-observer reliability, HP had shorter exam time and less invalid measurements. The study demonstrated well the correlations, diagnosis agreement and reliability of HP, a novel visual transient elastography comparing to FS, a well validated method in AP and LS measurement.

1) Although there is moderate correlations between two devices, the cutoffs in specific clinical scenario might be different. For FS, the cutoff of 10 and 15kPa for rule-out and rule-in cACLD were well accepted. However, the cutoff in hepatic steatosis diagnosis might be 248, 275 or 280 dB/m. Instead of grading steatosis, it might be useful to evaluate HP performance using different AP cutoffs by FS.

We thank the reviewer for this valuable suggestion. We agree that because Hepatus and FibroScan do not generate identical attenuation parameter values, the optimal attenuation parameter thresholds for Hepatus may differ from those established for FibroScan.

In the present study, our primary objective was to evaluate agreement between the two devices rather than derive device-specific diagnostic thresholds. Therefore, we used established FibroScan attenuation parameter cutoffs (248, 268, and 280 dB/m) to assess agreement in steatosis classification between the two modalities. We agree that determination of Hepatus-specific attenuation parameter thresholds represents an important area for future investigation and would ideally require validation against an independent reference standard such as MRI-PDFF or liver histology.

2) Was invalid measurements equal to failure rate? Was there difference in measurement reliability for viral and MASLD patients for HP device?

We thank the reviewer for this question. Invalid measurements were not equivalent to examination failure. An invalid measurement refers to an unsuccessful individual acquisition during an examination, whereas examination failure was defined as the inability to obtain at least 10 valid measurements after 30 attempts. Although FibroScan produced a higher number of invalid measurements compared with Hepatus, all examinations in both groups ultimately achieved the required number of valid measurements and therefore the overall examination success rate was 100% for both devices. We have clarified this distinction in the revised manuscript.

(page 13 line 263-266)
