# Supplementary material for: Comparing attenuation parameter and liver stiffness measurement using Hepatus and Fibroscan for the evaluation of steatosis and fibrosis in patients with chronic liver disease: A prospective study
Source: PLoS One. 2026 Nov 5;21(11):e0359831. doi: 10.1371/journal.pone.0359831 (PMC13739124; doi:10.1371/journal.pone.0359831)
Supplement: Supplementary file 5 [file pone.0359831.s007.docx]

**Reviewers' Comments to Author:**

**Reviewer 1:**

Thank you for submitting the revised manuscript. I sincerely appreciate the time and effort you invested in addressing my comments. Unfortunately, I regret that I cannot provide a more favorable assessment, as I do not believe that the major scientific and clinical concerns I previously raised have been adequately addressed.

**Response:**

We thank the reviewer for carefully evaluating our revised manuscript and for reiterating these important concerns. We appreciate that our previous revision did not sufficiently address the distinction between agreement with FibroScan and independent validation of Hepatus, and that additional analyses using available serum biomarkers could further strengthen the study.

In response, we have substantially revised the manuscript. Specifically, we have (1) further clarified that the present study is a comparative agreement and reproducibility study and does not establish the diagnostic accuracy of Hepatus against independent reference standards; (2) revised the Abstract, Discussion and Conclusion to avoid statements that could be interpreted as establishing diagnostic validation; and (3) performed additional exploratory patient-level analyses examining the relationships between Hepatus and FibroScan measurements and available serum biochemical markers, including AST, ALT and GGT.

**Comment 1**

Even if Hepatus demonstrates a correlation with FibroScan measurements, this does not necessarily indicate a direct correlation with the actual degree of liver fibrosis or steatosis.

**Response**

We agree with the reviewer that correlation and agreement between Hepatus and FibroScan should not be interpreted as establishing the diagnostic accuracy of Hepatus for the actual degree of liver fibrosis or steatosis. The objective of this study was to compare attenuation parameter and liver stiffness measurements obtained using Hepatus with FibroScan, together with their reproducibility and technical performance. Although FibroScan is an extensively validated and widely used non-invasive elastography platform, it remains an indirect assessment and therefore does not constitute an independent reference standard.

We have revised the manuscript to clearly distinguish agreement with FibroScan from diagnostic validation and have modified the Abstract, Discussion, Conclusion, and Limitations accordingly. We now explicitly acknowledge that further validation against independent reference standards, such as liver histology, MRE, or MRI-PDFF, is required to establish the diagnostic accuracy and device-specific thresholds of Hepatus.

**Changes made in the manuscript:** Abstract, Discussion, Conclusion and Limitations sections have been revised accordingly.

**Comment 2**

In addition, correlation analyses should be performed between both FibroScan and Hepatus measurements and established non-invasive serum biomarkers (e.g., AST, ALT, and other liver function markers) as well as validated non-invasive indices, such as the FIB-4 index.

**Response**

We thank the reviewer for reiterating this suggestion. In response, we performed additional exploratory patient-level correlations between liver stiffness measurements (LSM) obtained using Hepatus and FibroScan and the available serum biochemical markers. To account for repeated examinations, the four LSM measurements obtained with each device were averaged for each participant, and correlations were assessed using Spearman's rank correlation.

AST correlated significantly with LSM measured using Hepatus (rho = 0.297, p < 0.001) and FibroScan (rho = 0.381, p < 0.001). Similar correlations were observed for ALT (Hepatus: rho = 0.201, p = 0.012; FibroScan: rho = 0.251, p = 0.002) and GGT (Hepatus: rho = 0.320, p < 0.001; FibroScan: rho = 0.416, p < 0.001). These findings are now presented in Supplementary Table S3.

Correlations with attenuation parameter were not performed because routine serum biochemical markers are not validated measures of hepatic steatosis; assessment against established reference standards such as MRI-PDFF or liver histology would be more appropriate. We also acknowledge that the observed biochemical correlations with LSM are modest and should not be interpreted as diagnostic validation.

FIB-4 could not be calculated because platelet count was not collected in the original study dataset. This has been explicitly acknowledged as a limitation, and further validation using established fibrosis indices and independent histological or imaging reference standards is warranted.

**Changes made in the manuscript:** The Statistical Analysis, Results and Limitations sections have been revised, and Supplementary Table S3 has been added.

**Response to the Academic Editor**

A correlation analyses could be performed between both FibroScan and Hepatus measurements and established non-invasive serum biomarkers (e.g., AST, ALT, and other liver function markers) as well as validated non-invasive indices, such as the FIB-4 index. Or the point could be listed as one of the study limitations.

**Response:**

We thank the Academic Editor for this suggestion. In response, we performed additional exploratory patient-level correlation analyses between Hepatus and FibroScan measurements and available serum biochemical markers, including AST, ALT and GGT.

Significant positive correlations were observed between AST, ALT and GGT and liver stiffness measurements obtained using both Hepatus and FibroScan. These findings have now been incorporated into the Results and are presented in detail in Supplementary Table S3.

FIB-4 could not be calculated because platelet count was not collected in the original study dataset. We have explicitly acknowledged this limitation in the revised manuscript and have clarified that routine serum biochemical markers are not independent reference standards for fibrosis or steatosis.

**Data availability**

**Reviewer assessment:**

Have the authors made all data underlying the findings in their manuscript fully available? — No.

**Response:**

We thank the reviewer for highlighting this issue. We have now provided a de-identified participant-level dataset as Supporting Information (S1 Dataset) containing the data underlying the findings reported in the manuscript, including the repeated Hepatus and FibroScan measurements and variables required to reproduce the reported analyses. Direct patient identifiers have been removed to protect participant confidentiality.
