# Supplementary material for: Comparing attenuation parameter and liver stiffness measurement using Hepatus and Fibroscan for the evaluation of steatosis and fibrosis in patients with chronic liver disease: A prospective study
Source: PLoS One. 2026 Nov 5;21(11):e0359831. doi: 10.1371/journal.pone.0359831 (PMC13739124; doi:10.1371/journal.pone.0359831)
Supplement: Peer review history

# Peer review: Decision Letter 0

**DOI:** 10.1371/journal.pone.0359831.r001

**Authors:** Jee-Fu Huang

2 Jun 2026

Dear Dr. Chan,

Please submit your revised manuscript by Jul 17 2026 11:59PM. If you will need more time than this to complete your revisions, please reply to this message or contact the journal office at plosone@plos.org. When you're ready to submit your revision, log on to https://www.editorialmanager.com/pone/ and select the 'Submissions Needing Revision' folder to locate your manuscript file.

- A letter that responds to each point raised by the academic editor and reviewer(s). You should upload this letter as a separate file labeled 'Response to Reviewers'.
- A marked-up copy of your manuscript that highlights changes made to the original version. You should upload this as a separate file labeled 'Revised Manuscript with Track Changes'.
- An unmarked version of your revised paper without tracked changes. You should upload this as a separate file labeled 'Manuscript'.

If you would like to make changes to your financial disclosure, please include your updated statement in your cover letter. Guidelines for resubmitting your figure files are available below the reviewer comments at the end of this letter.

If applicable, we recommend that you deposit your laboratory protocols in protocols.io to enhance the reproducibility of your results. Protocols.io assigns your protocol its own identifier (DOI) so that it can be cited independently in the future. For instructions see: https://journals.plos.org/plosone/s/submission-guidelines#loc-laboratory-protocols. Additionally, PLOS ONE offers an option for publishing peer-reviewed Lab Protocol articles, which describe protocols hosted on protocols.io. Read more information on sharing protocols at https://plos.org/protocols?utm_medium=editorial-email&utm_source=authorletters&utm_campaign=protocols.

As the corresponding author, your ORCID iD is verified in the submission system and will appear in the published article. PLOS supports the use of ORCID, and we encourage all coauthors to register for an ORCID iD and use it as well. Please encourage your coauthors to verify their ORCID iD within the submission system before final acceptance, as unverified ORCID iDs will not appear in the published article. *Only*  the individual author can complete the verification step; PLOS staff *cannot*  verify ORCID iDs on behalf of authors.

We look forward to receiving your revised manuscript.

Kind regards,

Jee-Fu Huang, M.D., Ph.D.

Academic Editor

PLOS One

**Journal Requirements:**

1. When submitting your revision, we need you to address these additional requirements.

Please ensure that your manuscript meets PLOS ONE's style requirements, including those for file naming. The PLOS ONE style templates can be found at

https://journals.plos.org/plosone/s/file?id=wjVg/PLOSOne_formatting_sample_main_body.pdf and

https://journals.plos.org/plosone/s/file?id=ba62/PLOSOne_formatting_sample_title_authors_affiliations.pdf

2. Thank you for stating the following in the Acknowledgments Section of your manuscript:

We would like to thank Infinity Medical Sdn Bhd for providing the Hepatus device used in this study. The company was not involved in the study design; in the collection, analysis and interpretation of data; in the write up of the report; and in the decision to submit the paper for publication.

We note that you have provided funding information that is not currently declared in your Funding Statement. However, funding information should not appear in the Acknowledgments section or other areas of your manuscript. We will only publish funding information present in the Funding Statement section of the online submission form.

Please remove any funding-related text from the manuscript and let us know how you would like to update your Funding Statement. Currently, your Funding Statement reads as follows:

Infinity Medical Sdn Bhd provide the Hepatus device used in this study. The company was not involved in the study design; in the collection, analysis and interpretation of data; in the write up of the report; and in the decision to submit the paper for publication.

Please include your amended statements within your cover letter; we will change the online submission form on your behalf.

3. In the online submission form, you indicated that [The data that support the findings of this study are available from the corresponding author upon reasonable request].

All PLOS journals now require all data underlying the findings described in their manuscript to be freely available to other researchers, either a. In a public repository, b. Within the manuscript itself, or c. Uploaded as supplementary information.

This policy applies to all data except where public deposition would breach compliance with the protocol approved by your research ethics board. If your data cannot be made publicly available for ethical or legal reasons (e.g., public availability would compromise patient privacy), please explain your reasons on resubmission and your exemption request will be escalated for approval.

4. Please upload a new copy of Figures 1, 2 and 3, as the detail is not clear. Please follow the link for more information:  https://journals.plos.org/plosone/s/figures

5. If the reviewer comments include a recommendation to cite specific previously published works, please review and evaluate these publications to determine whether they are relevant and should be cited. There is no requirement to cite these works unless the editor has indicated otherwise.

[Note: HTML markup is below. Please do not edit.]

Reviewers' comments:

**Comments to the Author**

1. Is the manuscript technically sound, and do the data support the conclusions?

Reviewer #1: Partly

Reviewer #2: Yes

**********

2. Has the statistical analysis been performed appropriately and rigorously?

Reviewer #1: No

Reviewer #2: Yes

**********

3. Have the authors made all data underlying the findings in their manuscript fully available?

The PLOS Data policy

Reviewer #1: No

Reviewer #2: Yes

**********

4. Is the manuscript presented in an intelligible fashion and written in standard English?

Reviewer #1: Yes

Reviewer #2: Yes

**********

**Reviewer #1:**  This manuscript presents a comparison between Hepatus, a relatively new transient elastography device, and FibroScan for the assessment of hepatic steatosis and fibrosis in patients with chronic liver disease. The study addresses a clinically relevant topic, as the development of reliable and accessible non-invasive tools for liver disease assessment remains an important unmet need. The authors should be commended for the substantial effort involved in conducting repeated examinations by different operators and evaluating both intra- and inter-observer reliability. However, several major concerns regarding the study design, statistical analysis, and validation strategy limit the interpretation of the findings. Most importantly, the handling of repeated measurements raises concerns regarding the independence of observations, and the comparison with FibroScan alone is insufficient to establish the clinical validity of Hepatus as a tool for assessing liver fibrosis and steatosis. Furthermore, additional analyses are required to better characterize the performance of Hepatus across clinically relevant subgroups and in relation to established non-invasive biomarkers. Therefore, I recommend Major Revision. My specific comments are provided below.

1) This manuscript analyzes 1,264 examinations performed in 158 individuals. This corresponds to eight examinations per subject, assuming that two examiners each performed two different tests twice. While the substantial effort involved in collecting these data is commendable, there are statistical concerns regarding the treatment of all examination results as independent observations. Measurements obtained repeatedly from the same individual should not be considered independent variables; rather, they should be averaged or otherwise appropriately accounted for as repeated measures. In addition, the reproducibility of the measurements obtained from repeated examinations in the same patient should be evaluated and reported.

2) FibroScan has been extensively validated against established reference standards, including liver biopsy, for the assessment of hepatic fibrosis and steatosis. In contrast, as the authors themselves acknowledge, Hepatus is a relatively new technique for which robust clinical evidence has not yet been established. Furthermore, FibroScan provides only an indirect assessment of liver fibrosis and steatosis. Therefore, even if Hepatus demonstrates a correlation with FibroScan measurements, this does not necessarily indicate a direct correlation with the actual degree of fibrosis or steatosis. Before comparisons with FibroScan can be considered sufficient validation, the relationship between Hepatus and established reference standards, such as liver biopsy, MRI elastography, and MR spectroscopy, should be investigated.

3) The correlation between Hepatus and FibroScan measurements should be evaluated separately in relevant subgroups, including obese versus non-obese individuals and patients with viral versus non-viral liver disease.

4) In addition, correlation analyses should be performed between both FibroScan and Hepatus measurements and established non-invasive serum biomarkers (AST, ALT and so on) and indices, such as the FIB-4 index. Such analyses would provide further insight into the clinical validity and utility of these modalities for the assessment of liver fibrosis.

**Reviewer #2:**  General comments

This study compares attenuation parameter (AP) and liver stiffness (LS) measurements with Fibroscan (FS) and Hepatus (HP). The authors enrolled 158 chronic liver disease patients. Two operators in different backgrounds performed twice for each patient at two different points. The authors analyzed 1264 exams and showed moderate correlations in AP and LS between FS and HP. The mean difference of AP and LS were15 (-69-99) dB/m and 1.2 (-5.0-7.2 ) kPa. There were also agreement in the steatosis grade and diagnosis of compensated advanced chronic liver disease (cACLD). While both HP and FS were in good intra- and inter-observer reliability, HP had shorter exam time and less invalid measurements. The study demonstrated well the correlations, diagnosis agreement and reliability of HP, a novel visual transient elastography comparing to FS, a well validated method in AP and LS measurement.

Major comments

1. Although there is moderate correlations between two devices, the cutoffs in specific clinical scenario might be different. For FS, the cutoff of 10 and 15kPa for rule-out and rule-in cACLD were well accepted. However, the cutoff in hepatic steatosis diagnosis might be 248, 275 or 280 dB/m. Instead of grading steatosis, it might be useful to evaluate HP performance using different AP cutoffs by FS.

2. Was invalid measurements equal to failure rate? Was there difference in measurement reliability for viral and MASLD patients for HP device?

**********

what does this mean?). If published, this will include your full peer review and any attached files.

If you choose “no”, your identity will remain anonymous but your review may still be made public.

**Do you want your identity to be public for this peer review?**  For information about this choice, including consent withdrawal, please see our Privacy Policy

Reviewer #1: No

Reviewer #2: No

**********

[NOTE: If reviewer comments were submitted as an attachment file, they will be attached to this email and accessible via the submission site. Please log into your account, locate the manuscript record, and check for the action link "View Attachments". If this link does not appear, there are no attachment files.]

To ensure your figures meet our technical requirements, please review our figure guidelines: https://journals.plos.org/plosone/s/figures

You may also use PLOS’s free figure tool, NAAS, to help you prepare publication quality figures: https://journals.plos.org/plosone/s/figures#loc-tools-for-figure-preparation.

NAAS will assess whether your figures meet our technical requirements by comparing each figure against our figure specifications.

# Author comment: Author response to Decision Letter 1

**DOI:** 10.1371/journal.pone.0359831.r002

22 Jul 2026

We thank the Academic Editor and reviewers for their constructive comments and suggestions. We have carefully addressed each comment and revised the manuscript accordingly. A detailed point-by-point response to all reviewer and editor comments has been provided in the attached Response to Reviewers document.

Major revisions include clarification of the study design and statistical analysis, additional subgroup analyses, revisions to the Abstract, Methods, Results, Discussion, and Limitations sections, updated Funding and Data Availability Statements, and submission of higher-resolution figures as requested.

Attachment Submitted filename: Response_Reviewers_HS_FS_final.docx

# Peer review: Decision Letter 1

**DOI:** 10.1371/journal.pone.0359831.r003

**Authors:** Jee-Fu Huang

4 Aug 2026

Dear Dr. Chan,

Thank you for submitting your manuscript to PLOS ONE. After careful consideration, we feel that it has merit but does not fully meet PLOS ONE’s publication criteria as it currently stands. Therefore, we invite you to submit a revised version of the manuscript that addresses the points raised during the review process.

Please submit your revised manuscript by Sep 18 2026 11:59PM. If you will need more time than this to complete your revisions, please reply to this message or contact the journal office at plosone@plos.org. When you're ready to submit your revision, log on to https://www.editorialmanager.com/pone/ and select the 'Submissions Needing Revision' folder to locate your manuscript file.

Please include the following items when submitting your revised manuscript:

- A letter that responds to each point raised by the academic editor and reviewer(s). You should upload this letter as a separate file labeled 'Response to Reviewers'.
- A marked-up copy of your manuscript that highlights changes made to the original version. You should upload this as a separate file labeled 'Revised Manuscript with Track Changes'.
- An unmarked version of your revised paper without tracked changes. You should upload this as a separate file labeled 'Manuscript'.

If you would like to make changes to your financial disclosure, please include your updated statement in your cover letter. Guidelines for resubmitting your figure files are available below the reviewer comments at the end of this letter.

If applicable, we recommend that you deposit your laboratory protocols in protocols.io to enhance the reproducibility of your results. Protocols.io assigns your protocol its own identifier (DOI) so that it can be cited independently in the future. For instructions see: https://journals.plos.org/plosone/s/submission-guidelines#loc-laboratory-protocols. Additionally, PLOS ONE offers an option for publishing peer-reviewed Lab Protocol articles, which describe protocols hosted on protocols.io. Read more information on sharing protocols at https://plos.org/protocols?utm_medium=editorial-email&utm_source=authorletters&utm_campaign=protocols.

As the corresponding author, your ORCID iD is verified in the submission system and will appear in the published article. PLOS supports the use of ORCID, and we encourage all coauthors to register for an ORCID iD and use it as well. Please encourage your coauthors to verify their ORCID iD within the submission system before final acceptance, as unverified ORCID iDs will not appear in the published article. Only the individual author can complete the verification step; PLOS staff cannot verify ORCID iDs on behalf of authors.

We look forward to receiving your revised manuscript.

Kind regards,

Jee-Fu Huang, M.D., Ph.D.

Academic Editor

PLOS One

Journal Requirements:

If the reviewer comments include a recommendation to cite specific previously published works, please review and evaluate these publications to determine whether they are relevant and should be cited. There is no requirement to cite these works unless the editor has indicated otherwise.

Please review your reference list to ensure that it is complete and correct. If you have cited papers that have been retracted, please include the rationale for doing so in the manuscript text, or remove these references and replace them with relevant current references. Any changes to the reference list should be mentioned in the rebuttal letter that accompanies your revised manuscript. If you need to cite a retracted article, indicate the article’s retracted status in the References list and also include a citation and full reference for the retraction notice.

**Additional Editor Comments:**

A correlation analyses could be performed between both FibroScan and Hepatus measurements and established non-invasive serum biomarkers (e.g., AST, ALT, and other liver function markers) as well as validated non-invasive indices, such as the FIB-4 index. Or the point could be listed as one of the study limitations.

[Note: HTML markup is below. Please do not edit.]

Reviewers' comments:

Reviewer's Responses to Questions

**Comments to the Author**

Reviewer #1: (No Response)

Reviewer #2: All comments have been addressed

**********

2. Is the manuscript technically sound, and do the data support the conclusions?

Reviewer #1: No

Reviewer #2: (No Response)

**********

3. Has the statistical analysis been performed appropriately and rigorously?

Reviewer #1: Yes

Reviewer #2: (No Response)

**********

4. Have the authors made all data underlying the findings in their manuscript fully available?

The PLOS Data policy

Reviewer #1: No

Reviewer #2: (No Response)

**********

5. Is the manuscript presented in an intelligible fashion and written in standard English?

Reviewer #1: Yes

Reviewer #2: (No Response)

**********

Reviewer #1: Thank you for submitting the revised manuscript. I sincerely appreciate the time and effort you invested in addressing my comments. Unfortunately, I regret that I cannot provide a more favorable assessment, as I do not believe that the major scientific and clinical concerns I previously raised have been adequately addressed.

In particular, I consider the lack of clear responses to the following two issues to be a critical weakness that precludes publication of the manuscript.

1) Even if Hepatus demonstrates a correlation with FibroScan measurements, this does not necessarily indicate a direct correlation with the actual degree of liver fibrosis or steatosis.

2) In addition, correlation analyses should be performed between both FibroScan and Hepatus measurements and established non-invasive serum biomarkers (e.g., AST, ALT, and other liver function markers) as well as validated non-invasive indices, such as the FIB-4 index.

Reviewer #2: (No Response)

**********

what does this mean?). If published, this will include your full peer review and any attached files.

If you choose “no”, your identity will remain anonymous but your review may still be made public.

**Do you want your identity to be public for this peer review?**  For information about this choice, including consent withdrawal, please see our Privacy Policy

Reviewer #1: No

Reviewer #2: No

**********

[NOTE: If reviewer comments were submitted as an attachment file, they will be attached to this email and accessible via the submission site. Please log into your account, locate the manuscript record, and check for the action link "View Attachments". If this link does not appear, there are no attachment files.]

To ensure your figures meet our technical requirements, please review our figure guidelines: https://journals.plos.org/plosone/s/figures

You may also use PLOS’s free figure tool, NAAS, to help you prepare publication quality figures: https://journals.plos.org/plosone/s/figures#loc-tools-for-figure-preparation.

NAAS will assess whether your figures meet our technical requirements by comparing each figure against our figure specifications.

# Author comment: Author response to Decision Letter 2

**DOI:** 10.1371/journal.pone.0359831.r004

6 Sep 2026

We thank the Academic Editor and reviewers for their careful evaluation of our revised manuscript. We have addressed the remaining comments as detailed below.

Reviewer 1:

Thank you for submitting the revised manuscript. I sincerely appreciate the time and effort you invested in addressing my comments. Unfortunately, I regret that I cannot provide a more favorable assessment, as I do not believe that the major scientific and clinical concerns I previously raised have been adequately addressed.

Response:

We thank the reviewer for carefully evaluating our revised manuscript and for reiterating these important concerns. We appreciate that our previous revision did not sufficiently address the distinction between agreement with FibroScan and independent validation of Hepatus, and that additional analyses using available serum biomarkers could further strengthen the study.

In response, we have substantially revised the manuscript. Specifically, we have (1) further clarified that the present study is a comparative agreement and reproducibility study and does not establish the diagnostic accuracy of Hepatus against independent reference standards; (2) revised the Abstract, Discussion and Conclusion to avoid statements that could be interpreted as establishing diagnostic validation; and (3) performed additional exploratory patient-level analyses examining the relationships between Hepatus and FibroScan measurements and available serum biochemical markers, including AST, ALT and GGT.

Comment 1

Even if Hepatus demonstrates a correlation with FibroScan measurements, this does not necessarily indicate a direct correlation with the actual degree of liver fibrosis or steatosis.

Response

We agree with the reviewer that correlation and agreement between Hepatus and FibroScan should not be interpreted as establishing the diagnostic accuracy of Hepatus for the actual degree of liver fibrosis or steatosis. The objective of this study was to compare attenuation parameter and liver stiffness measurements obtained using Hepatus with FibroScan, together with their reproducibility and technical performance. Although FibroScan is an extensively validated and widely used non-invasive elastography platform, it remains an indirect assessment and therefore does not constitute an independent reference standard.

We have revised the manuscript to clearly distinguish agreement with FibroScan from diagnostic validation and have modified the Abstract, Discussion, Conclusion, and Limitations accordingly. We now explicitly acknowledge that further validation against independent reference standards, such as liver histology, MRE, or MRI-PDFF, is required to establish the diagnostic accuracy and device-specific thresholds of Hepatus.

Changes made in the manuscript: Abstract, Discussion, Conclusion and Limitations sections have been revised accordingly.

Comment 2

In addition, correlation analyses should be performed between both FibroScan and Hepatus measurements and established non-invasive serum biomarkers (e.g., AST, ALT, and other liver function markers) as well as validated non-invasive indices, such as the FIB-4 index.

Response

We thank the reviewer for reiterating this suggestion. In response, we performed additional exploratory patient-level correlations between liver stiffness measurements (LSM) obtained using Hepatus and FibroScan and the available serum biochemical markers. To account for repeated examinations, the four LSM measurements obtained with each device were averaged for each participant, and correlations were assessed using Spearman's rank correlation.

AST correlated significantly with LSM measured using Hepatus (rho = 0.297, p < 0.001) and FibroScan (rho = 0.381, p < 0.001). Similar correlations were observed for ALT (Hepatus: rho = 0.201, p = 0.012; FibroScan: rho = 0.251, p = 0.002) and GGT (Hepatus: rho = 0.320, p < 0.001; FibroScan: rho = 0.416, p < 0.001). These findings are now presented in Supplementary Table S3.

Correlations with attenuation parameter were not performed because routine serum biochemical markers are not validated measures of hepatic steatosis; assessment against established reference standards such as MRI-PDFF or liver histology would be more appropriate. We also acknowledge that the observed biochemical correlations with LSM are modest and should not be interpreted as diagnostic validation.

FIB-4 could not be calculated because platelet count was not collected in the original study dataset. This has been explicitly acknowledged as a limitation, and further validation using established fibrosis indices and independent histological or imaging reference standards is warranted.

Changes made in the manuscript: The Statistical Analysis, Results and Limitations sections have been revised, and Supplementary Table S3 has been added.

Response to the Academic Editor

A correlation analyses could be performed between both FibroScan and Hepatus measurements and established non-invasive serum biomarkers (e.g., AST, ALT, and other liver function markers) as well as validated non-invasive indices, such as the FIB-4 index. Or the point could be listed as one of the study limitations.

Response:

We thank the Academic Editor for this suggestion. In response, we performed additional exploratory patient-level correlation analyses between Hepatus and FibroScan measurements and available serum biochemical markers, including AST, ALT and GGT.

Significant positive correlations were observed between AST, ALT and GGT and liver stiffness measurements obtained using both Hepatus and FibroScan. These findings have now been incorporated into the Results and are presented in detail in Supplementary Table S3.

FIB-4 could not be calculated because platelet count was not collected in the original study dataset. We have explicitly acknowledged this limitation in the revised manuscript and have clarified that routine serum biochemical markers are not independent reference standards for fibrosis or steatosis.

Data availability

Reviewer assessment:

Have the authors made all data underlying the findings in their manuscript fully available? — No.

Response:

We thank the reviewer for highlighting this issue. We have now provided a de-identified participant-level dataset as Supporting Information (S1 Dataset) containing the data underlying the findings reported in the manuscript, including the repeated Hepatus and FibroScan measurements and variables required to reproduce the reported analyses. Direct patient identifiers have been removed to protect participant confidentiality.

We hope that these revisions have satisfactorily addressed the remaining concerns of the Academic Editor and reviewer.

Attachment Submitted filename: Reviewers_response.docx

# Peer review: Decision Letter 2

**DOI:** 10.1371/journal.pone.0359831.r005

**Authors:** Jee-Fu Huang

21 Sep 2026

<p>Comparing attenuation parameter and liver stiffness measurement using Hepatus and Fibroscan for the evaluation of steatosis and fibrosis in patients with chronic liver disease: a prospective study

PONE-D-26-20472R2

Dear Dr. Chan,

We’re pleased to inform you that your manuscript has been judged scientifically suitable for publication and will be formally accepted for publication once it meets all outstanding technical requirements.

Within one week, you’ll receive an e-mail detailing the required amendments. When these have been addressed, you’ll receive a formal acceptance letter and your manuscript will be scheduled for publication.

An invoice will be generated when your article is formally accepted. Please note, if your institution has a publishing partnership with PLOS and your article meets the relevant criteria, all or part of your publication costs will be covered. Please make sure your user information is up-to-date by logging into Editorial Manager at Editorial Manager® and clicking the ‘Update My Information' link at the top of the page. For questions related to billing, please contact billing support.

If your institution or institutions have a press office, please notify them about your upcoming paper to help maximize its impact. If they’ll be preparing press materials, please inform our press team as soon as possible -- no later than 48 hours after receiving the formal acceptance. Your manuscript will remain under strict press embargo until 2 pm Eastern Time on the date of publication. For more information, please contact onepress@plos.org.

Kind regards,

Jee-Fu Huang, M.D., Ph.D.

Academic Editor

PLOS One

Additional Editor Comments (optional):

Reviewers' comments:

Reviewer's Responses to Questions

**Comments to the Author**

Reviewer #1: (No Response)

**********

2. Is the manuscript technically sound, and do the data support the conclusions?

Reviewer #1: Yes

**********

3. Has the statistical analysis been performed appropriately and rigorously?

Reviewer #1: Yes

**********

4. Have the authors made all data underlying the findings in their manuscript fully available?

The PLOS Data policy

Reviewer #1: Yes

**********

5. Is the manuscript presented in an intelligible fashion and written in standard English?

Reviewer #1: Yes

**********

Reviewer #1: Thank you for submitting the revised manuscript. I considered the authors’ responses and understand their explanations. I have no further comments or concerns regarding the manuscript.

**********

what does this mean?). If published, this will include your full peer review and any attached files.

If you choose “no”, your identity will remain anonymous but your review may still be made public.

**Do you want your identity to be public for this peer review?**  For information about this choice, including consent withdrawal, please see our Privacy Policy

Reviewer #1: No

**********

# Editor report: Acceptance letter

**DOI:** 10.1371/journal.pone.0359831.r006

**Authors:** Jee-Fu Huang

PONE-D-26-20472R2

PLOS One

Dear Dr. Chan,

I'm pleased to inform you that your manuscript has been deemed suitable for publication in PLOS One. Congratulations! Your manuscript is now being handed over to our production team.

At this stage, our production department will prepare your paper for publication. This includes ensuring the following:

* All references, tables, and figures are properly cited

* All relevant supporting information is included in the manuscript submission,

* There are no issues that prevent the paper from being properly typeset

You will receive further instructions from the production team, including instructions on how to review your proof when it is ready. Please keep in mind that we are working through a large volume of accepted articles, so please give us a few days to review your paper and let you know the next and final steps.

Lastly, if your institution or institutions have a press office, please let them know about your upcoming paper now to help maximize its impact. If they'll be preparing press materials, please inform our press team within the next 48 hours. Your manuscript will remain under strict press embargo until 2 pm Eastern Time on the date of publication. For more information, please contact onepress@plos.org.

You will receive an invoice from PLOS for your publication fee after your manuscript has reached the completed accept phase. If you receive an email requesting payment before acceptance or for any other service, this may be a phishing scheme. Learn how to identify phishing emails and protect your accounts at https://explore.plos.org/phishing.

If we can help with anything else, please email us at customercare@plos.org.

Thank you for submitting your work to PLOS One and supporting open access.

Kind regards,

PLOS One Editorial Office Staff

on behalf of

Dr. Jee-Fu Huang

Academic Editor

PLOS One
